# Wnt/β-catenin Signalling Is Active in a Highly Dynamic Pattern during Development of the Mouse Cerebellum

**DOI:** 10.1371/journal.pone.0023012

**Published:** 2011-08-08

**Authors:** Hayden J. Selvadurai, John O. Mason

**Affiliations:** Centre for Integrative Physiology, School of Biomedical Sciences, University of Edinburgh, Edinburgh, United Kingdom; Northwestern University Feinberg School of Medicine, United States of America

## Abstract

The adult cerebellum is composed of several distinct cell types with well defined developmental origins. However, the molecular mechanisms that govern the generation of these cell types are only partially resolved. Wnt/β-catenin signalling has a wide variety of roles in generation of the central nervous system, though the specific activity of this pathway during cerebellum development is not well understood. Here, we present data that delineate the spatio-temporal specific pattern of Wnt/β-catenin signaling during mouse cerebellum development between E12.5 and P21. Using the BAT-gal Wnt/β-catenin reporter mouse, we found that Wnt/β-catenin activity is present transiently at the embryonic rhombic lip but not at later stages during the expansion of cell populations that arise from there. At late embryonic and early postnatal stages, Wnt/β-catenin activity shifts to the cerebellar ventricular zone and to cells arising from this germinal centre. Subsequently, the expression pattern becomes progressively restricted to Bergmann glial cells, which show expression of the reporter at P21. These results indicate a variety of potential functions for Wnt/β-catenin activity during cerebellum development.

## Introduction

The cerebellum forms as a result of a highly regulated programme of cell specification, proliferation, differentiation and migration (reviewed in [1]). At the cellular level, the cerebellum is organised into distinct neuronal layers: the outermost molecular layer (ML), the Purkinje cell monolayer (PCL), the densely populated internal granule layer (IGL) and the innermost white matter (WM). The diverse cell types that make up these layers originate from two distinct germinal centres in the early cerebellum; the ventricular zone - a monolayer of cells lining the fourth ventricle on the ventral surface of the cerebellar anlage, and the rhombic lip - a transient structure in the most posterior part of the cerebellar anlage that forms the interface between the neural tube and non-neural roofplate ectoderm (reviewed in [2]).

The rhombic lip gives rise to the entire complement of glutamatergic neurons that populate the IGL. The first glutamatergic neurons born are the projection neurons of the deep cerebellar nuclei (DCN). In the mouse these arise between embryonic day (E) 10.5 and E12.5 [3], and migrate along the sub pial stream to the rostral end of the developing cerebellum. From E12.5 onwards, the rhombic lip generates granule progenitor cells (GPCs) and unipolar brush cells (UBCs) [4], [5], [6]. Exiting the rhombic lip, GPCs migrate rostrally across the pial surface of the cerebellum to form a secondary germinal zone, the external germinal layer (EGL), which covers the pial surface of the cerebellum. This cell layer proliferates extensively until the second postnatal week, producing a vast number of mature granule cells (GCs), which become post-mitotic within the EGL before migrating radially along Bergmann glial fibres into the IGL, a process that is complete by postnatal day (P) 21 [1], [7], [8].

Distinct from the rhombic lip, the ventricular zone gives rise to all cerebellar cells of the γ-aminobutyric acid (GABA)ergic, and glial lineages. The first of these, the Purkinje cells, are born from E10.5 then migrate radially towards the pial surface of the cerebellum and settle as a distinct monolayer of cells around the time of birth (E19.5-E20) [9]. Closely following this the Bergmann glia are generated and migrate radially behind the developing Purkinje cell population before undergoing morphological maturation postnatally [10], [11]. Interneurons (including stellate, basket, Golgi and Lugaro interneurons) and the remaining glial population (velate and fibrous astrocytes) are then generated in a sequential manner. These cell types are derived from progenitors that delaminate from the ventricular zone and continue to divide in the WM [12], [13], [14], [15], [16], [17].

These tightly coordinated developmental processes rely on the spatio-temporal specific activity of several key signalling pathways. The sonic hedgehog (Shh) signalling pathway, for example, is the main mitogenic factor driving GPC proliferation within the EGL [18], [19]. The Wnt/β-catenin signalling pathway has been shown to play an important part in regulation of neural stem and progenitor populations within the central nervous system [20], [21], but its role in cerebellum development is only partially defined. Wnt1 is an important regulator of early cerebellum development. It is expressed at the isthmus and rhombic lip [22], [23], [24], [25], and in cells of the granule lineage [26] and *Wnt1^−/−^* mutant mice completely lack or exhibit a severely underdeveloped cerebellum [27], [28], [29]. No specific role for Wnt/β-catenin signalling in later stages of cerebellum development has yet been described. However, activating mutations in components of the Wnt/β-catenin signalling pathway have been identified in medulloblastomas, a paediatric tumour that arises in the posterior fossa (cerebellum plus brainstem) [30], [31]. Further, individuals carrying germline mutations in the tumour suppressor gene *APC* that constitutively activate Wnt/β-catenin signalling have a greatly elevated risk of developing medullobastoma [32]. This raises the possibility that Wnt/β-catenin signalling regulates developmental processes in the cerebellum, and that activation of this pathway may cause these processes to go awry, predisposing to the development of medulloblastoma (reviewed in [33]). Recent *in vivo* evidence supports this [34].

In order to investigate possible roles played by Wnt/β-catenin signalling during development of the cerebellum, we used the BAT-gal reporter transgenic mouse strain [35] to identify regions of the developing cerebellum where Wnt/β-catenin signalling is active. At mid-gestational stages, we found active Wnt/β-catenin signalling in the rhombic lip. At perinatal stages, activity was seen in the ventricular zone, but not in the EGL. Postnatally, signalling became progressively more restricted, and very few proliferating cells expressed the BAT-gal transgene. Surprisingly, we found that many of the Wnt/β-catenin responsive cells in the postnatal cerebellum were Bergmann glia.

## Results

### Wnt/β-catenin signalling is active at the isthmus and rhombic lip

**Figure 1:**
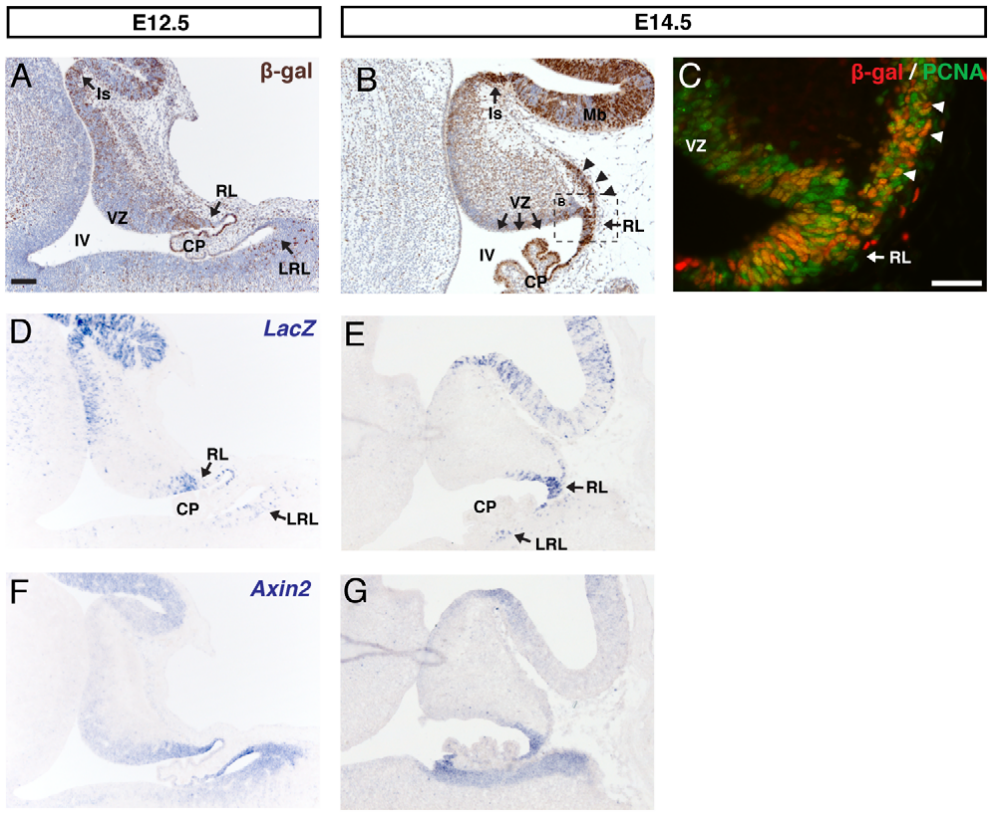
BAT-gal expression in the E12.5 and E14.5 cerebellum. (A) DAB Immunohistochemistry for β-galactosidase (β-gal) on sagittal sections of E12.5 cerebellum revealed two key expression domains: the isthmus (Is) and the cerebellar rhombic lip (RL). At E14.5 (B) expression was also found in the early external granule layer (EGL, black arrowheads) but was notably absent from the ventricular zone (VZ) lining the fourth ventricle (IV). (C) Double immunofluorescence for β-gal and PCNA confirms the expression of β-gal in the RL and EGL (white arrowheads). β-gal protein was validated as a Wnt/β-catenin reporter by *in situ* hybridisation for *LacZ* (D, E) and Wnt target *Axin2* (F, G)) mRNA. At both time points β-gal protein and *LacZ* mRNA were expressed in the same domains as *Axin2*, although the *LacZ* mRNA expression appeared less diffuse than that of *Axin2*. (A–B counterstained with hematoxylin. Scale bars: A, B, D–E  = 100 µm, B  = 50 µM).

The BAT-gal transgenic reporter strain expresses a *lacZ* gene under the control of β-catenin/T cell factor (TCF) responsive elements [35] and has been widely used as a general reporter of Wnt/β-catenin activity. We first examined the expression of β-galactosidase (the protein product of the *LacZ* transgene) from E12.5 to E14.5 in cerebellum sections from BAT-gal+ embryos. β-galactosidase expression was observed in a progressively restricted pattern (Fig. 1). Firstly, at E12.5 expression was observed at the isthmus in the anterior region of the cerebellum and at the rhombic lip forming the posterior region of the cerebellum (Fig. 1A). In addition β-galactosidase expressing (β-gal+) cells were observed spread diffusely through the anterior and posterior regions of the cerebellum, although were notably sparse within the ventricular zone.

In contrast, at E14.5 β-galactosidase expression was more restricted to the anterior and posterior ends of the cerebellum. While β-gal+ cells were absent from the ventricular zone, they were observed clearly at the rhombic lip (Fig. 1B). Staining for proliferating cell nuclear antigen (PCNA), which marks proliferating cells including those in the rhombic lip area, confirmed the presence of β-gal+ cells at the rhombic lip and within the population of early GPCs that have begun to migrate in an anterior direction along the pial surface of the cerebellum (arrowheads in Fig. 1B–C).

Since β-galactosidase protein is a stable protein and can persist in tissue after *lacZ* gene expression ceases [36], we also examined expression of *lacZ* mRNA by in situ hybridization (Fig. 1D–E). The expression pattern found was very similar to that observed by immunohistochemistry (compare Fig. 1A to 1D and 1B to 1E), although there are slight differences that would suggest the β-galactosidase protein expression may to some extent label cells that are no longer responding to a Wnt/β-catenin signal. For example, β-galactosidase protein expression was found in the dorsal aspect of the choroid plexus, whereas the expression of *LacZ* mRNA is restricted to the rhombic lip. Importantly however, the key β-gal protein expression domains identified (rhombic lip and isthmus) mirror those of the *LacZ* expression.

As an independent verification that BAT-gal reporter expression in the developing cerebellum truly indicates Wnt/β-catenin activity we also performed *in situ* hybridisation for *Axin2*. *Axin2* encodes a negative feedback inhibitor of the Wnt/β-catenin signalling pathway. It is a direct target of TCF/LEF-mediated transcription and is therefore widely used as a readout of Wnt/β-catenin signalling [37]. Within the isthmus and the rhombic lip, the expression of *Axin2* (Fig. 1F–G) closely mirrored both *LacZ* mRNA expression (Fig. 1D–E) and β-galactosidase protein expression (Fig. 1A–B). However, at E12.5, *Axin2* expression was not detected in the anterior portion of the cerebellum immediately below the isthmus (Fig. 1F). At E12.5 and E14.5, *Axin2* showed more diffuse expression in a gradient from both the upper and the lower rhombic lips (Fig. 1F–G), compared to both *LacZ* staining and β-galactosidase protein expression.

### Wnt/β-catenin signalling is active in the ventricular zone but not in the EGL of the perinatal cerebellum

**Figure 2:**
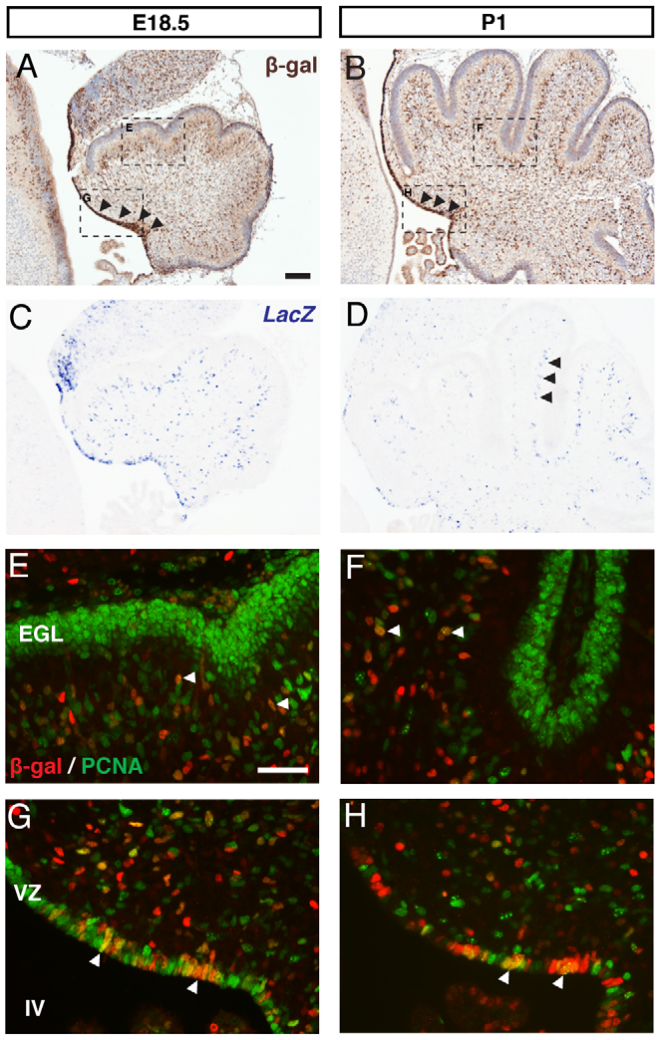
BAT-gal expression in the E18.5 and P1 cerebellum. DAB immunohistochemistry for β-gal in sagittal sections of the E18.5 cerebellum (A) reveals widespread expression, including predominant staining in the VZ (black arrowheads). A similar pattern is observed at P1 (B). These expression patterns are mirrored by those observed for *LacZ* mRNA visualised with in situ hybridisation (C–D). Double immunofluorescence for β-gal and PCNA reveals an almost complete lack of BAT-gal reporter expression in the EGL at both E18.5 (E) and P1 (F), though β-gal+ cells can be observed within the developing cerebellum at both time points, in some cases colocalised with PCNA (white arrows). At the VZ, BAT-gal expression can be observed colocalised with PCNA (white arrowheads) at both E18.5 (G) and P1 (H). (A–B counterstained with hematoxylin. Scale bars: A, B = 100 µm, E–H = 50 µm).

We next examined BAT-gal reporter expression at two perinatal stages, E18.5 and P1. In contrast to the highly restricted expression of β-galactosidase seen at E14.5, we found much more widespread expression at these time points (Fig. 2). β-gal+ cells were spread through several developing cell layers of the cerebellum, though were notably absent from the EGL (Fig. 2A–B). This pattern is also observed in the expression of *LacZ* (Fig. 2C–D), indicating that perdurance of β-galactosidase is not a significant issue at these ages.

While the rhombic lip and early migratory GPCs showed abundant expression of β-galactosidase at E14.5, the absence of detectable β-galactosidase expression within the EGL of the perinatal cerebellum suggests that Wnt/β-catenin signalling is not involved in the continued development of this cell population. PCNA staining clearly labels the EGL at E18.5 and P1 and co-staining for β-galactosidase clearly supported the lack of β-gal+ cells in the EGL at these stages (Fig. 2E–F).

An abundance of β-gal+ cells were identified in the cerebellar ventricular zone at E18.5 (Fig. 2A) and P1 (Fig. 2B), in contrast to E14.5. PCNA labelling clearly delineates the ventricular zone at these stages, visible as a thin layer of cells lining the fourth ventricle. Double staining for β-galactosidase and PCNA confirmed the presence of proliferative β-gal+ cells within the ventricular zone (arrowheads in Fig. 2G–H). Interestingly, by P1 there were a number of β-gal+ cells that did not express PCNA, indicative of a non-proliferative cell type. β-gal+ cells were also found within the developing cerebellar anlage, consistent with cells migrating from the progenitor monolayer (Fig. 2E–H). Some of these were PCNA-positive (arrowheads in Fig. 2E–F).

### Wnt/β-catenin signalling becomes increasingly restricted and does not correlate with proliferation during postnatal development

**Figure 3:**
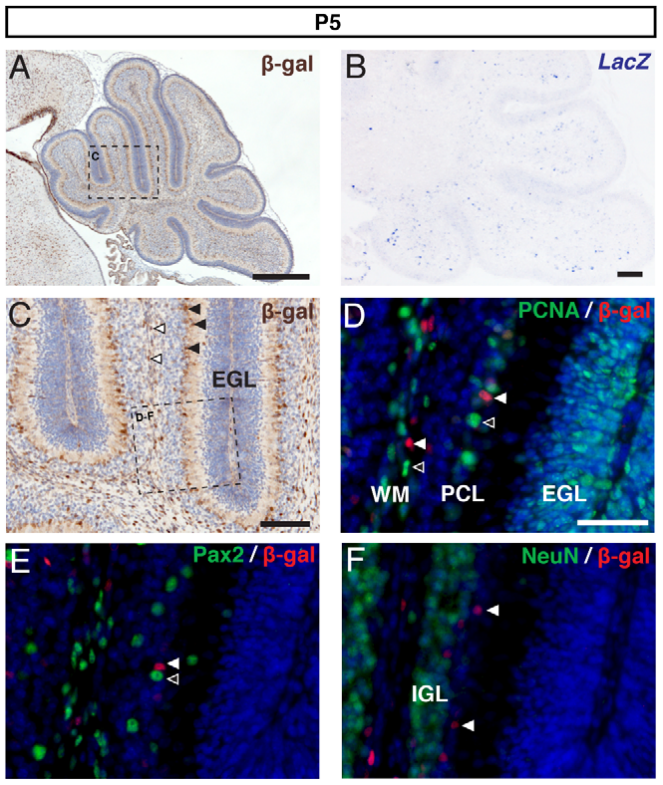
BAT-gal expression in the P5 cerebellum. (A) DAB immunohistochemistry for β-gal and (B) *LacZ* in situ hybridisation in the P5 cerebellum. (C) Higher magnification of the region boxed in (A) reveals expression spread through all layers except the EGL. The Purkinje cell layer (PCL) and the white matter (WM) in particular contained many β-gal+ cells (black and white arrowheads respectively). Double immunofluorescence for β-gal and PCNA (D) revealed the presence of β-gal+ cells within the PCL and white matter (white arrowheads). Although β-gal+ cells were observed in close proximity to proliferating cells (unfilled arrowheads) very few β-gal+/PCNA+ cells were observed. Double immunofluorescence for β-gal and Pax2 (E) showed the close proximity of β-gal+ cells (white arrowhead) to Pax2+ interneurons (unfilled arrowhead) but no double-labelled cells were observed. Double immunofluorescence for β-gal and NeuN showed β-gal+ cells (white arrows) located outwith the IGL. (A,C counterstained with hematoxylin and D–F with Topro3. Scale bars: A = 500 µm, B–C = 100 µm, D–E = 50 µm).

**Figure 4:**
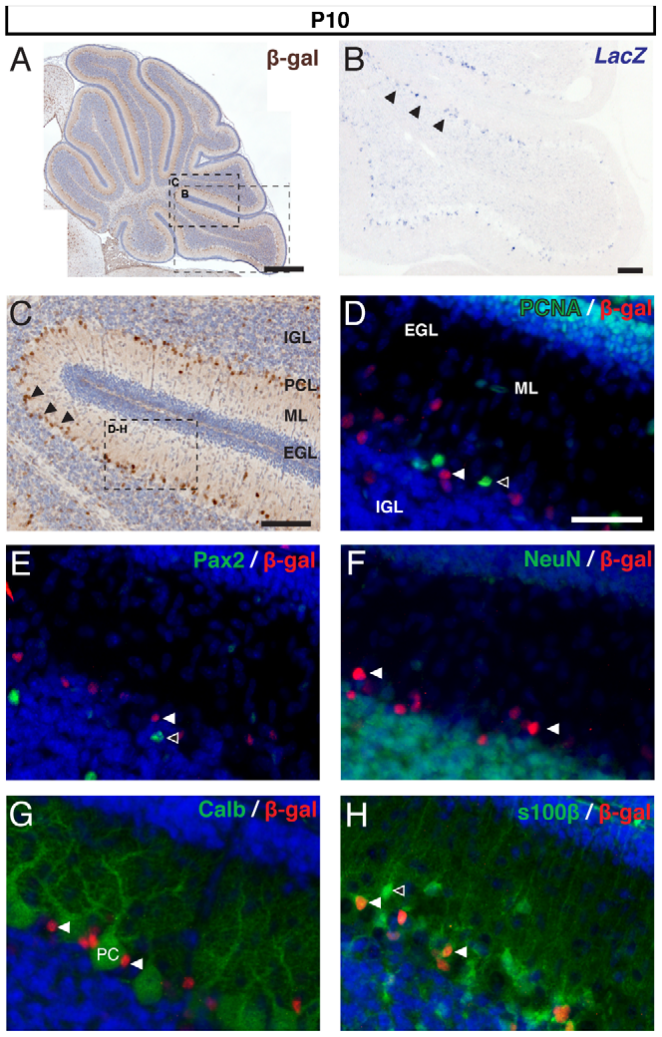
BAT-gal expression in the P10 cerebellum. (A) DAB β-gal immunohistochemistry and (B) *LacZ* in situ hybridisation in P10 cerebellum. (C) Higher magnification of the region boxed in (A) reveals a more restricted pattern than that seen at P5, with strongest staining observed within the PCL (black arrowheads – also in B). At higher magnification, β-gal+ cells within the PCL (white arrowheads) were observed in close proximity to both PCNA+ (D) and Pax2+ (E) cells (unfilled arrowheads), though no colocalisation was observed between β-gal and PCNA or Pax2. (F) Double immunofluorescence for β-gal and NeuN confirms the presence of β-gal+ cells at the PCL on the edge of the IGL, while double immunofluorescence for β-gal and calbindin (G) confirms the lack of BAT-gal reporter expression in Purkinje cells (PC). (H) Colocalisation with glial marker s100β confirms the identity of β-gal+ cells within the PCL as Bergmann glia (white arrowheads), though not all Bergmann glia express β-galactosidase (unfilled arrowhead). (A, C are counterstained with hematoxylin and D–H with Topro3 Scale bars: A = 500 µm, B–C = 100 µm, D–H = 50 µm).

**Figure 5:**
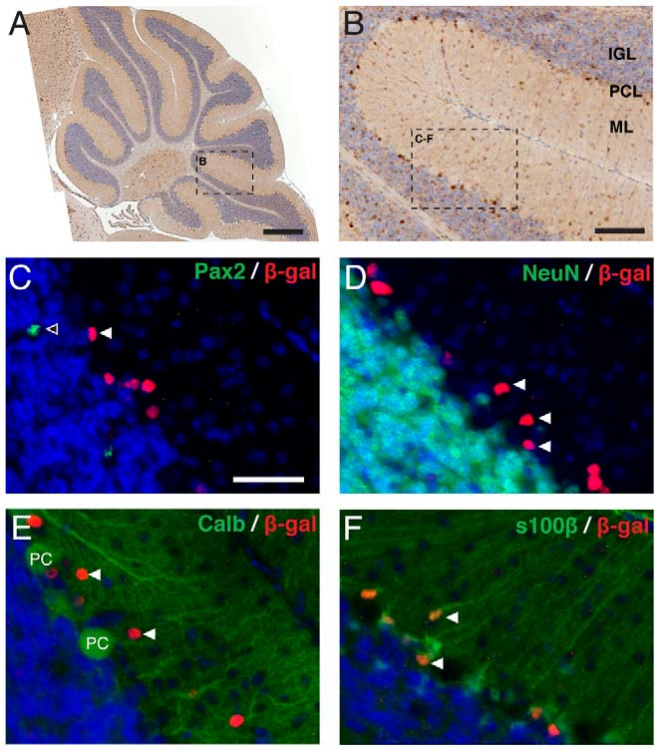
BAT-gal expression in the P21 cerebellum. (A) DAB immunohistochemistry for β-gal in the P21 cerebellum. (B) Higher magnification of the region boxed in (A) reveals that BAT-gal reporter expression is largely restricted to the PCL (black arrowheads), with few β-gal+ cells observed in other layers. Double immunofluorescence experiments confirmed this localisation of β-gal+ cells (C–F). Double immunofluorescence for Pax2 (C), NeuN (D) and Calbindin (E) show the β-gal+ cells (white arrows) located in the PCL do not express markers of interneurons, granule neurons or PCs respectively. (F) Colocalisation with glial marker s100β confirms the identity of these cells as Bergmann glia (white arrows). (A–B are counterstained with hematoxylin and C–F with Topro3. Scale bars: A = 500 µm, B = 100 µm, C–F = 50 µm).

We next examined patterns of BAT-gal reporter expression at later postnatal stages. By P5, all of the layers that make up the mature cerebellum (WM, IGL, PCL, ML) can be identified - along with the transient EGL. We found β-gal+ cells in each of these layers, except for the EGL (Fig. 3A–B). A similar pattern was observed at P10 (Fig. 4A–B), although β-gal+ cells were most abundant within the PCL and WM at this stage. Increasingly restricted distribution was also seen at P21, by which time β-gal+ cells were largely restricted to the PCL (Fig. 5A–B). This pattern was also observed in the expression of *LacZ* mRNA (Fig. 3B, 4B), further illustrating that β-galactosidase immunohistochemistry accurately reflects expression of the BAT-gal transgene till P10. However, by P21 there was no identifiable expression of *LacZ* mRNA (data not shown) indicating that the β-gal+ cell types identified at that late stage may no longer be responding to a Wnt/β-catenin signal and could be maintaining detectable levels of the β-galactosidase protein from when they last transduced a Wnt/β-catenin signal.

As Wnt/β-catenin signalling is commonly associated with the control of cell proliferation, we wanted to determine whether the BAT-gal expression pattern correlated with proliferation between P5 and P10 (the most highly proliferative period of cerebellar development). We therefore performed double immunofluorescence staining for β-galactosidase and PCNA. A number of PCNA+ cells were seen in each layer at P5 (Fig. 3D), becoming restricted to the EGL, WM and PCL by P10 (Fig. 4D) reflecting the overall decrease in proliferation by this point. By P21 PCNA+ cells were largely restricted to the WM (data not shown), reflecting the proliferation of white matter progenitors. While β-gal+ cells were identified in all these key regions, very few β-gal+/PCNA+ cells were seen at P5 (Fig. 3D), and none were identified at P10 (Fig. 4D) or P21 (data not shown). Thus, we found little evidence for correlation between Wnt/β-catenin signalling and proliferation in the postnatal cerebellum, despite the identification of many β-gal+ cells in proliferative regions such as the WM and PCL.

### Wnt/β-catenin signalling is seen in Bergmann glia in the postnatal cerebellum

We next sought to identify the specific cell type(s) within the postnatal cerebellum in which Wnt/β-catenin signalling is active. The locations of the β-gal+ cell population identified at each postnatal stage examined suggested a number of possible cell types, including interneuron and glial progenitors within the WM, granule cells, astrocytes, oligodendrocytes and interneurons within the IGL, Purkinje cells and Bergmann glia within the PCL and interneurons within the ML. To determine the identity of the β-gal+ cells we performed double immunofluorescence experiments between β-galactosidase and a number of marker proteins known to label specific cerebellar cell types.

The transcription factor Pax2 is expressed in committed cerebellar interneurons after their exit from the ventricular zone and prior to their terminal differentiation [15]. Thus, colocalisation between Pax2 and β-galactosidase would indicate that committed interneuron progenitors were responding to a Wnt/β-catenin signal. The pattern of Pax2 expression identified at all three stages analysed was consistent with that expected for interneuron precursors. However, although many β-gal+ and Pax2+ cells were observed in the same cell layers, often in close proximity to each other, no colocalisation was observed in any sections analysed (Fig. 3E, 4E, 5C).

The β-gal+ cells in the IGL, PCL and ML could also be migratory post-mitotic granule cells, exiting the EGL towards their final destination in the IGL. However, double immunofluorescence between β-galactosidase and NeuN, a marker for post-mitotic granule cells [38], did not reveal any colocalisation of the two proteins in any sections analysed (Fig. 3F, 4F, 5D). Thus, Wnt/β-catenin signalling appears unlikely to be directly involved in the migration of post-mitotic granule cells.

Many β-gal+ cells were clearly localised to the PCL, suggesting the possibility that Purkinje cells may be responding to Wnt/β-catenin signalling. However, no colocalisation was observed between β-galactosidase and calbindin, a marker that clearly identifies Purkinje cells from P10, at either P10 (Fig. 4G) or P21 (Fig. 5E). Interestingly, many of the β-gal+ cells in the PCL were in close proximity to Purkinje cells, consistent with the location of Bergmann glia.

To determine whether Wnt/β-catenin signalling indeed marks a population of glial cells, we performed double immunofluorescence with s100β, a marker for Bergman glia and other astrocytes from P10 [39]. As expected, we identified β-gal+/s100β+ cells in the PCL at P10 (Fig. 4H) and P21 (Fig. 5F) consistent with the conclusion that a population of Bergman glia respond to a Wnt/β-catenin signal during development.

## Discussion

**Figure 6:**
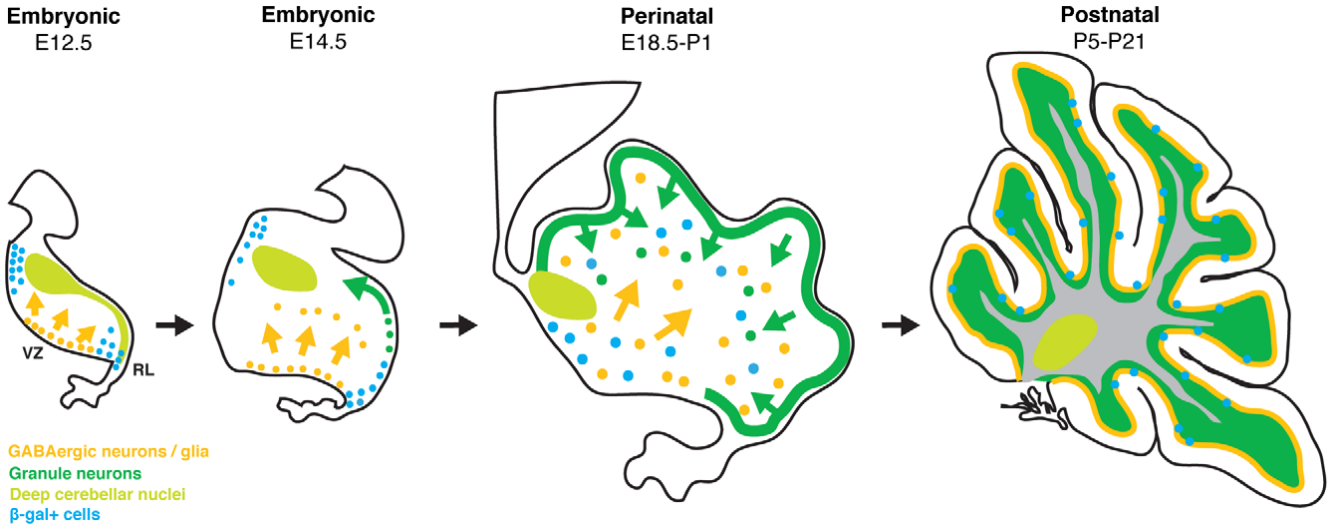
Summary of Wntβ-catenin signalling during cerebellum development. Wnt/β-catenin signalling is present in a dynamic spatio-temporal specific pattern in the developing cerebellum. Initially it is observed at the cerebellar rhombic lip but by E18.5 its expression expands into a more widespread pattern with particularly strong expression at the VZ during the birth of glia and interneurons. During postnatal development it is largely restricted to the PCL, consistent with a subpopulation of Bergmann glia.

In this study we have investigated the distribution of Wnt/β-catenin signalling during development of the cerebellum from E12.5 to P21 primarily using the BAT-gal Wnt reporter mouse strain [35]. The specific roles played by Wnt/β-catenin signalling during development of the cerebellum are not yet well characterised. Here, we provide evidence for a specific and dynamic spatio-temporal pattern of Wnt/β-catenin signalling through different stages of cerebellum development (summarised in Fig. 6).

### Wnt/β-catenin signalling is active at the rhombic lip but not expansion or differentiation of GPCs

Our experiments revealed expression of the BAT-gal reporter at the rhombic lip at E12.5 and early EGL at E14.5. The rhombic lip gives birth to projection neurons of the deep cerebellar nuclei from E10.5 to E12.5 [3] followed by GPCs and unipolar brush cells from E12.5 onwards [4], [5], [6]. Because unipolar brush cells migrate along a different path than the dorsal stream that forms the EGL, we conclude that the BAT-gal reporter expression observed at E12.5 and E14.5 is potentially limited to GPCs and late born DCN neurons.

Consistent with this, a number of studies have identified expression of *Wnt1* at the rhombic lip and at the isthmus [22], [23], [24], [25], [26] and loss of *Wnt1* leads to a severe developmental phenotype of the cerebellum, most likely due to a failure to maintain the isthmus [27], [28], [29]. Due to the consistency between the known expression pattern of *Wnt1*, and its proven role as a key signalling molecule in this area, it is possible that Wnt1 activity is responsible for the active Wnt/β-catenin signalling at the embryonic isthmus and rhombic lip identified in our experiments. However, it remains to be established whether additional Wnt genes are expressed in this area.

While active Wnt/β-catenin signalling was observed in the early migrating GPCs at E14.5 (Fig. 1A–B), this was lost in the GPCs observed in the EGL during later stages of development. By E18.5, BAT-gal expression within the EGL was minimal and from P1 onwards, it was undetectable (Fig. 2A–D). These data are consistent with a potential role for Wnt/β-catenin signalling during early specification of GPCs but not in their further migration or proliferation. This is consistent with the fact that proliferation of this cell population during late embryogenesis and early postnatal development is driven by Sonic hedgehog secreted by neighbouring Purkinje cells [18], [19].

Additionally, the absence of NeuN expression in any of the β-gal+ cells observed from P5-P21 demonstrates that Wnt/β-catenin signalling is also not active in the migration of terminally differentiated GCs from the EGL to the IGL. (Fig. 3E, 4E, 5D). NeuN is abundantly expressed in most classes of neurons [38] and has been identified in all stages of post-mitotic granule cell development [40]. Thus, the lack of NeuN expression in β-gal+ cells located in these regions indicates that they are not of the granule lineage.

### A potential role for Wnt/β-catenin signalling in development of cell lineages from the cerebellar ventricular zone

At E18.5 and P1, many β-gal+ cells were seen in the ventricular zone (Fig. 2), although none were found there earlier (Fig. 1). This indicates that Wnt/β-catenin signalling is active in cell lineages originating at the ventricular zone at these time points. Purkinje cells arise at the onset of cerebellar neurogenesis between E10.5 and E12.5 [8], [9]. The interneuron lineage can then be detected by the expression of Pax2 in scattered cells at the ventricular zone from E13.5 to E17.5 [15] from where they migrate radially as Pax2+ lineage restricted progenitors [14], [15]. Concurrently, gliogenesis begins at the ventricular zone from E13.5, identified through the expression of S100β, BLBP and Sox9 [41]. The early Bergmann glial population exits the ventricular zone at E14.5 and follows a migratory path behind Purkinje cells [10]. Birth of the remaining cerebellar glial populations (astrocytes and oligodendrocytes) follows from this point. Our data suggest that the cell populations born at the ventricular zone between E18.5 and P1 could be doing so in response to a Wnt/β-catenin signal. Based upon the timing of known cell populations arising from the ventricular zone, this is most likely limited to glial and interneuron progenitors.

### Wnt/β-catenin signalling may persist in the glial population throughout postnatal cerebellum development

Further to the identification of β-gal+ cells at the ventricular zone, we also observed a population of β-gal+ cells within all other layers of the cerebellum (excluding the EGL) from E18.5 through to P21. Lack of colocalisation between β-galactosidase and Pax2 (Fig. 3E, 4E, 5C), NeuN (Fig. 3F, 4F, 5D) and Calbindin (Fig. 4G, 5E) ruled out the possibility of the β-gal+ cell population being of the interneuron, granule or Purkinje cell types respectively.

The remaining alternative is that the β-gal+ cell population identified within the developing cerebellum are glia. Oligodendrocytes are thought to arise from extra-cerebellar tissue [42], while velate and fibrous astrocytes arise from ventricular zone derived WM progenitor cells. Bergmann glia are thought to follow a slightly different developmental path. Rather than arising during gliogenesis from WM progenitors like the rest of the astrocyte lineage, a population of early Bergmann glia arise from the ventricular zone and migrate in close proximity to - and remain developmentally intertwined with - Purkinje cells [10]. This wave of migration occurs from E14.5 onwards, and by E18.5 these glia come to lie in a pattern similar to that seen for some of the β-gal+ cell population we identified, outlining the developing folia inferior to the EGL. A β-gal+ cell population in this pattern was seen at all postnatal stages, though the number of labelled cells appears to decrease with age. While limitations of the antibody used mean we were unable to confirm the identity of this cell population prior to P5, colocalisation of β-galactosidase with s100β in cells present in close proximity to Purkinje cells at both P10 (Fig. 4G) and P21 (Fig. 5F) supports the hypothesis that some Bergmann glia respond to a Wnt/β-catenin signal during development. Expression of *LacZ* mRNA identified at all stages except for P21 supports a potential role for Wnt/β-catenin signalling during development, and would suggest that residual β-galactosidase protein has been identified at P21.

Interestingly, the lack of β-galactosidase expression at the ventricular zone at E14.5 indicates that Wnt/β-catenin signalling is not involved in the birth of the Bergmann glia but may be potentially involved in its further development and maturation. This is consistent with the postnatal dynamic transformation of Bergmann glia alongside dendritogenesis and synaptogenesis of Purkinje cells [11] and suggests a possible role for Wnt/β-catenin in this process.

### Relevance to the developmental origins of medulloblastoma

We have shown that Wnt/β-catenin signalling is active in a highly dynamic and varied spatiotemporal pattern during key stages of cerebellum development. Surprisingly, Wnt/β-catenin is also active in a subset of Bergmann glia in the postnatal cerebellum. How these results relate to the development of medulloblastoma is confounded by recent findings by Gibson al [34]. These authors provide evidence that constitutive activation of the Wnt/β-catenin pathway in BLBP expressing cerebellar precursors causes a defect in cell migration from the lower rhombic lip - which manifests eventually as medulloblastoma if tumour suppressor TP53 is also deactivated - while cell populations arising from the ventricular zone and rhombic lip do not show evidence for any developmental defect. Interestingly, we found BAT-gal and *Axin2* expression in the lower rhombic lip (Fig. 1) supporting their conclusion that the lower rhombic lip is a Wnt responsive area. However, our findings that Wnt/β-catenin signalling activity is in the upper rhombic lip and in cells arising from the ventricular zone now warrants more in depth functional investigation to determine the role of this pathway in development of these cell populations.

## Methods

### Mice

The licence authorising this work was approved by the University of Edinburgh's Ethical Review Committee on 22nd September 2008 (application number PL35-08) and by the Home Office on 6th November 2008. Animal husbandry was in accordance with the UK Animals (Scientific Procedures) Act 1986 regulations. To minimise animal suffering, pregnant dams were culled by cervical dislocation under terminal anaesthesia according to the Code of Practice for Humane Killing of Animals under Schedule 1 to the Animals (Scientific Procedures) Act 1986 issued by the Home Office. The day the vaginal plug was detected was designated E0.5 and the day of birth as P0. BAT-gal mice were maintained on a C57BL/6J genetic background and were genotyped as described previously [35]. Wild type mice on the same background were obtained by crossing mice hemizygous for the BAT-gal transgene and used as negative controls.

### Histology

Embryos were collected at E12.5 and E18.5 and pups between P1 and P21. Whole E12.5 embryos, heads from E14.5 embryos and brains dissected from E18.5 embryos were immersion fixed in 4% paraformaldehyde (PFA) in phosphate buffered saline (PBS) overnight at 4°C. P1, 5, 10 and 21 pups were anaesthetised with Avertin and transcardially perfused with PFA, followed by tissue dissection and overnight immersion fixation in fresh PFA. Embryonic and postnatal tissue for immunohistochemistry and immunofluorescence were all processed following standard conditions [43], embedded in paraffin wax and cut in serial 10 µm sections on a sagittal plane. Tissue for *in situ* hybridisation was cryoprotected in 30% sucrose/PBS overnight before embedding in 30% sucrose/OCT (1∶1) and snap freezing. Frozen sections were cut on a cryostat at 14 µm. At least two animals were analysed at each age.

### Immunohistochemistry, immunofluorescence and *in situ* hybridisation

Immunohistochemistry and immunofluorescence were performed according to standard protocols. Antigen retrieval was achieved by microwaving sections at full power in 10 mM citrate buffer (pH6.0) for five minutes followed by 15 minutes at medium power. Primary antibodies were rabbit anti-β-galactosidase (Molecular Probes, 1∶1000 for immunohistochemistry, 1∶500 for immunofluorescence), mouse anti-β-galactosidase (DSHB, 1∶500), mouse anti-PCNA (Abcam, 1∶500), mouse anti-Pax2 (Covance, 1∶200), mouse anti-NeuN (Millipore, 1∶500), mouse anti-Calbindin (Swant, 1∶500), mouse anti-s100β (Abcam, 1∶500). Secondary non-fluorescent antibodies were biotinylated anti-rabbit IgG (Dako, 1∶200).

For single immunohistochemistry experiments the dark brown signal was revealed after incubation with the ABC kit (Vector), followed by a diaminobenzidine (DAB) and hydrogen peroxide reaction using the DAB detection kit (Vector). For double immunofluorescence experiments, all primary antibodies were detected using goat anti-rabbit IgG or goat anti-mouse IgG secondary antibodies conjugated to Alexa fluor 568 or 488 dyes (Invitrogen, 1∶200) respectively. Appropriate controls were used in all cases by incubating sections with all but the primary antibodies. No staining was observed under these conditions.

*In situ* hybridisation on frozen section was performed as described previously [44]. *LacZ* and *Axin2* antisense riboprobes [45] were labelled using the digoxigenin RNA labelling kit (Roche) according to the manufacturer's instructions.

### Microscopy

A Leica brightfield microscope connected to a Leica DFC 480 digital camera was used to capture images of DAB labelled sections. A Leica brightfield microscope connected to a Leica DFC360Fx camera was used to capture images of fluorescently labelled sections.
